# Adolescents’ understanding of chemotherapy-related adverse events: a concept elicitation study*

**DOI:** 10.1590/1518-8345.6245.3717

**Published:** 2022-11-07

**Authors:** Fernanda Machado Silva-Rodrigues, Pamela S. Hinds, Lucila Castanheira Nascimento

**Affiliations:** 1 Faculdade de Ciências Médicas da Santa Casa de São Paulo, São Paulo, SP, Brazil.; 2 Universidade de São Paulo, Escola de Enfermagem de Ribeirão Preto, PAHO/WHO Collaborating Centre for Nursing Research Development, Ribeirão Preto, SP, Brazil.; 4 Children’s National Hospital, Director of Nursing Research, Department of Nursing Science, Professional Practice & Quality, Washington, DC, United States of America.

**Keywords:** Neoplasms, Symptom Assessment, Antineoplastics Agents, Drug-Related Side Effects and Adverse Reactions, Adolescent, Oncology Nursing

## Abstract

**Objective:**

to document adolescents’ understanding of chemotherapy-related core adverse events from the Pediatric Patient-Reported Outcomes version of the Common Terminology Criteria for Adverse Events and thus begin the validation process of this tool’s items with Brazilian adolescents.

**Method:**

this is a prospective, qualitative study of concept elicitation. The participants were 17 adolescents aged 13-18 years and undergoing chemotherapy in three hospitals in São Paulo - SP, Brazil. Cognitive interviews were conducted with questions based on chemotherapy-related adverse events. Data were analyzed for responsiveness and missingness.

**Results:**

adolescents could and were willing to provide descriptive information about their chemotherapy adverse events, including physical and emotional events. Some participants suggested alternative terms to name the adverse events and some used more complex terms, but most were satisfied with the primary terms used by the researchers.

**Conclusion:**

this study represents the first steps towards understanding how adolescent cancer patients identify, name, and describe these events by cognitive interviewing to help design future assessment instruments focused on this age group.

## Introduction

Cancer in adolescents corresponds to 2 to 3% of cancers in general^1^. The World Health Organization (WHO) classifies adolescents as individuals aged 10-19 years. Similarly, the cancer literature considers this age group as individuals aged 10-18 years^2^ ^-^ ^3^. Adolescents tend to be a high-risk group due to delayed diagnosis and poorer adherence to treatment^2^ ^,^ ^4^ ^-^ ^5^. This age group is also known for its unique biological and psychological complexities that can characterize its experience with cancer treatment^6^.

According to the Brazilian National Cancer Institute (INCA), cancer represents the second most common cause of death in adolescents^7^ and includes various neoplasms common in children and adults^8^. The most common cancers among Brazilian adolescents aged 15-18 years are Hodgkin’s lymphoma, non-Hodgkin’s lymphoma, acute lymphocytic leukemia, acute myeloid leukemia, thyroid cancer, central nervous system tumors, sarcomas, melanoma, and ovarian cancer^7^ ^,^ ^9^.

Chemotherapy is the most frequently used treatment for cancer in children and adolescents. Because of their chemical compounds, the antineoplastics related to adverse events (AEs) affect the quality of life of adolescents in different ways, such as by psychological and emotional events, neuropsychological deficits, changes in daily activities performance, and lack of concentration^10^. WHO understands AEs as complications with many different natures, associated with the use of drugs or other interventions. However, to be considered an adverse event, the drug or intervention does not necessarily have a causal relationship with the observed episode^11^. The medical literature in oncology and the nomenclature of terms in pharmacovigilance understand that the global concept of AEs includes adverse effects, symptoms, and toxicities. For this study, we chose to use the term adverse event and its abbreviation AE according to the Common Terminology Criteria for Adverse Events (CTCAE), an official set of terms related to possible events associated with anticancer therapy in clinical trials. In this context, AEs are related to the drug used and its dosage and the ones mostly reported in adolescents are fatigue, loss of appetite, weight loss, nausea, sleep disturbances, pain, mood changes, and depression^12^.

Adolescents experience several adverse events related to chemotherapy, and research shows that they may experience more of these events and related distress than young children^2^ ^,^ ^4^ ^,^ ^13^. Inadequate assessment of these adverse events can lead to poor suffering management and decreased quality of life. The Brazilian literature lacks studies about adolescents’ experience undergoing chemotherapy, especially regarding the adverse events associated with this treatment. This study thus sought to document adolescents’ understanding of the chemotherapy-related core AEs from the Pediatric Patient-Reported Outcomes version of the Common Terminology Criteria for Adverse Events (Ped-PRO-CTCAE^®^) and begin the validation process of this tool’s items with Brazilian adolescents. 

## Method

### Study design

This prospective concept elicitation study used descriptive cognitive interviewing (CI) to explore adolescents’ understanding of the adverse events of chemotherapy. CI is essential to develop, to refine, or to validate questionnaires or measures^14^. It can be both reparative and descriptive^14^ ^-^ ^15^. The United States Food and Drug Administration (FDA) and the literature on measurement tools development recommend conducting qualitative interviews with specific populations to support item generation and the content validity of Patient-Reported Outcomes (PRO) instruments^16^.

Cognitive interviewing can also be conducted without a specific measurement tool or a questionnaire when it aims to understand the behavior, the interpretation, or the comprehension of a specific population or construct of interest, as intended in this study^17^. A free-form format of descriptive cognitive interviewing was adopted to explore the constructs and specific language that adolescents with cancer use to describe the adverse events of chemotherapy. Data were collected from medical records, including patients’ underlying disease, information about the protocol used, and phase of treatment during data collection. 

### Data collection

For this study, the terms referring to adverse effects extracted from the Pediatric PRO-CTCAE^®^ were translated into and adapted for Brazilian Portuguese and later validated by three specialists in pediatric oncology, who assessed the efficiency of the translation of terms and their correspondence according to the following classifications: 1. adequate; 2. partially adequate; 3. inadequate. When items were classified as 2 or 3, professionals were asked to suggest the changes they deemed appropriate. To check the reliability and agreement between the experts’ assessments, the Gwet AC2 coefficient was applied^18^. Agreement among experts was 86.8% (AC2 = 0.868), which indicated “almost perfect agreement” (0.81 - 1) according to the reference values for these coefficients^18^. The experts suggested changing the order of terms or omitting some words to simplify the names.

**Figure 1:**
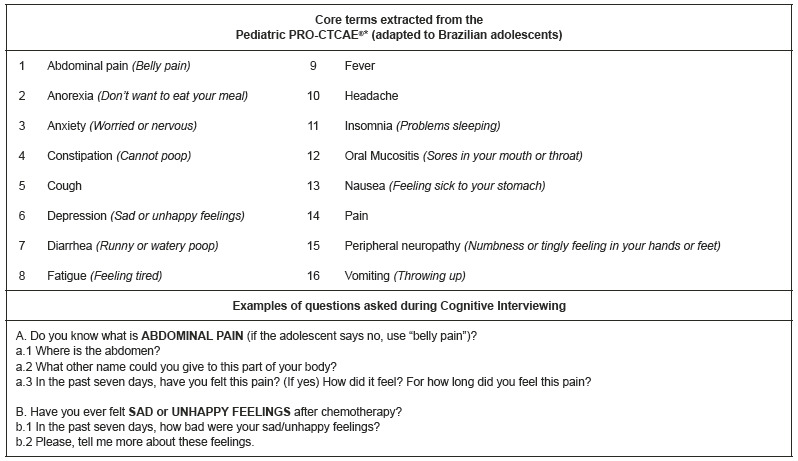
Core terms extracted from the Pediatric PRO-CTCAE^®*^ and examples of questions asked during the Cognitive Interviews. São Paulo, SP, Brazil, 2019 *Pediatric Patient-Reported Outcomes version of the Common Terminology Criteria for Adverse Events

A trained nurse researcher with no previous relationship with the participants interviewed them individually. The mean duration of the interviews was 16 minutes. The interview guide and the list of the core AEs were based on other similar studies^19^ ^-^ ^22^ to develop the Pediatric Patient-Reported Outcomes of the Common Terminology Criteria for Adverse Events (Pediatric PRO-CTCAE^®^) tool. The 16 core items include the most observed adverse events related to chemotherapy in children and adolescents. Figure 1 shows the core items and examples of questions that guided the cognitive interview. 

Questions targeted a seven-day recall period, same as prior pediatric cancer studies^21^ ^-^ ^23^. Initially the plan was to ask adolescents about four randomly selected AEs, but all participants showed readiness and interest to continue describing more events, with no signs of tiredness or apparent distraction. The mean duration of the interviews was 16 minutes.

### Participants and setting

Twenty-one adolescents were invited to join the study from December 2018 to March 2019. Before selecting the adolescents who met the study’s inclusion criteria, we consulted the staff nurses and patient records regarding patient eligibility and general clinical conditions. Three of the eligible patients declined to participate since they were feeling unwell at the time of the interview and another patient described feeling uncomfortable to answer the questions due to shyness. A purposive sample of 17 adolescents (aged 13-18) diagnosed with cancer and undergoing chemotherapy comprised the final study sample. The adolescents who presented cognitive impairments ‒ confirmed in their medical records and by their responsible health professionals ‒ were excluded. 

Data were collected at the pediatric oncology wards and outpatient clinics. A doctoral student conducted the interviews assisted by two research assistants, trained explicitly in probing questions and cognitive interviewing, under the supervision of a doctoral-prepared researcher with large experience in qualitative interviews. Some relevant clinical information was obtained from patients’ medical records, including details about the chemotherapy regimen and phase of treatment. Moreover, professionals responsible for the patients’ direct care were consulted about any restrictions that could preclude the adolescents’ participation in the study. 

The study was conducted in three pediatric hospitals: *Hospital Infantil Darcy Vargas* (HIDV), *Instituto de Oncologia Pediátrica do Grupo de Apoio ao Adolescente e à Criança com Câncer* (IOP - GRAACC), and *Serviço de Oncologia Pediátrica da Irmandade da Santa Casa de Misericórdia de São Paulo* (ISCMSP). All three are considered national high-complexity oncology centers located in São Paulo - SP, Brazil.

### Data analysis

After data collection, the interview was transcribed by two research assistants and validated by the main investigator. Accordingly to CI, verbal probing approaches were used to assess adolescents’ interpretations of each AE. The primary analysis was qualitative according to the content of phrases and the AE descriptions provided by adolescents, as recommended by the CI method^14^. The adolescents were also interviewed regarding their attributions and understanding of the events that preceded the AEs and the following outcomes.

### Ethical aspects

This study was approved by the Institutional Review Boards of the three centers involved (ethical approval numbers: *Certificado de Apresentação para Apreciação Ética* [CAAE]: 97412818.0.0000.5479/2.900.412 and CAAE: 97412818.0.3001.5505/3.117.534). All participants (adolescents and legal guardians) were given verbal and written information about the study and each provided informed assent/consent to participate.

## Results

The mean age of the 17 participants was 15 years, ranging from 13 to 18 years. Regarding gender, nine (53%) participants were female and eight (47%) were male. Six (35.4%) had leukemia as their primary diagnosis, five (29.4%) were diagnosed with bone tumors (osteosarcoma and sarcoma), three (17.6%) had lymphoma, and three (17,6%) presented other solid tumors. The adolescents’ mean time of treatment was 7.18 months. Two adolescents (11.8%) had relapsed disease. Participants were identified by the letter P, followed by a number.

We asked the adolescents about their interpretation of each AE and how they named them (if using any alternative terms). Probing questions were asked every time the adolescents mentioned any factors associated with the AEs to better explore preceding factors and possible outcomes associated with the events. All adolescents reviewed at least six AEs, with a mean of 8.41 AEs. They also provided autobiographical descriptions of adverse events they experienced during treatment, precisely distinguishing the events caused by the different drugs combined in the chemotherapy regimens.

### Physical AEs 

Gastrointestinal AEs were the most detailed physical events, including described duration, precipitating factors, and characteristics. To refer to nausea, the adolescents used the original term “nausea” and “feeling sick” instead of “sickness”: *Yes, I fell sick* (P6, 13 years old); *Nausea is the urge of vomiting, it’s when you want to puke* (P1, 14 years old). Adolescents discerned the adverse events related to the different anticancer drugs used: *It depends on the “chemo,” the worst is the red one* (P2, 14 years old); *The urge to vomit depends on the “chemo”* (P1, 14 years old). Nausea was therefore related to vomiting. Another participant reported nausea even before undergoing chemotherapy, known as anticipatory nausea: *I feel nausea before starting [chemo], which I think is psychological. I try to avoid anything that provokes vomit because if I think about, see or sniff it, I vomit* (P10, 16 years old).

They also associated constipation with chemotherapy, even when receiving other medications that affected gastrointestinal functions: *Yes, I was recently hospitalized for this. While I was receiving “chemo” at the hospital, I couldn’t poop* (P1, 14 years old). Constipation was related to abdominal pain and three participants (33.3%) used the term “abdominal pain” when asked if they would give any other name to “belly pain.” One of them stated having more than one type of abdominal pain, describing what could be considered visceral pain: *There are two types of pain in the stomach. The abdominal pain is when the muscles are aching, and the other is when your belly is hurting in the intestine* (P16, 15 years old). 

Overall, participants showed full ability to name pain sites and characteristics. One participant stated that, besides pain in the usual places, she felt generalized pain: *I’ve felt (pain) in both legs. Sometimes, in the belly and the body, like when you sleep uncomfortable. And other times, general pain in the whole body* (P2, 14 years old). One participant related pain to a particular drug: *If I’m not wrong, it was when I took MTX [Methotrexate]; I felt pain in the bones, in my ribs, it hurt a lot, and in my back too* (P16, 15 years old).

Other AEs, such as oral mucositis, caused the patients discomfort and interfered with eating: *It bothered me because my gums were swollen, and it was impossible to eat properly* (P15, 14 years old). The interview questions used the expression “sores that caused pain in the mouth and throat” rather than “oral mucositis”. Only one participant used the term “mucositis” when describing such lesions: *I had small mucositis and could hardly consider it mucositis. It was tiny, like a cold sore* (P16, 15 years old). One adolescent reported needing to receive analgesics to deal with mucositis: *The “chemo” gives us mucositis, and we cannot eat. I had to stay with morphine every hour because [I]) was full of wounds, and I couldn’t eat anything* (P10, 16 years old). 

Besides the inability to eat secondary to mucositis, participants also presented lack of appetite secondary to treatment: *I started taking “chemo” in October, and I lost even more weight. So far, I don’t feel hungry, but it has gotten better. I’m now 39 kg, and previously I was 46 kg* (P18, 16 years old).

Participants also described having fever and fatigue. They understood fatigue as “tiredness” that interfered with routine activities: *Yes [I felt tired], and I couldn’t do anything* (P5, 13 years old). One participant confirmed the fatigue interference: *I don’t even feel like walking. You get tired quickly, even while climbing stairs* (P12, 16 years old). Only one adolescent used the word “fatigue” when referring to this AE. 

Only one adolescent recognized cough as an AE, but according to his description, cough was related to a pulmonary condition not specifically associated to the chemotherapy treatment: *I had [cough], but it was because of the infection in the lung. Then, during the “chemo” period, I coughed due to an infection* (P10, 16 years old).

Regarding physical AEs, participants showed an understanding of the cause-and-effect relationship between chemotherapy drugs and AEs, identifying factors that could intensify the events. One adolescent, when asked about what could aggravate nausea, said: *Anything that reminds me of the hospital, like the food there, for example* (P7, 15 years old). Other examples of the cause-and-effect relation found were: *Tiredness comes when immunity is low* (P12, 16 years old); *Acidic food, like orange juice, aggravates the mucositis* (P17, 16 years old). Some participants also precisely described the duration of the events: *It happened in the last 21 days. We have 21 days to recover, and I think I’ll recover after these 21 days* (P17, 16 years old). Sometimes adolescents used temporal references: *It lasted all day* (P20, 15 years old); *it lasted for two or three weeks* (P7, 15 years old).

### Feelings and emotional AEs

The cognitive interviews included emotional changes such as sadness and unhappy feelings related to chemotherapy. All participants showed understanding of the term “depression” and some used it spontaneously when talking about sadness. One of the interviewees said chemotherapy made her more sensitive: *I noticed that I’ve become more sensitive because of any little thing since I started receiving chemotherapy* (P6, 13 years old).

Another participant emphasized the understanding of the relationship between these feelings and the chemotherapy treatment: *Sometimes I go home, get in a bad mood, and cry a lot. All of a sudden, I get sad, I start to cry. I didn’t want to do anything these days, and I just wanted to sit still. I asked my mother to leave me alone, then she left, and I cried* (P20, 15 years old).

Another adolescent used the word “depression” when referring to sad feelings related to chemotherapy, along with discouragement to perform some activities: *I think it’s depression and that there are several types of it. I feel deep sadness, and I don’t feel like doing anything* (P1, 14 years old). A boy attributed his sadness to the shock of hair loss and its impact on his self-esteem, a critical situational factor to consider: *Oh, I was shocked because I like to fix and change my hair. Then, they said it [hair] was going to fall out. I thought: Oh, I’m going bald, the girls won’t look at me anymore [he laughs]* (P7, 15 years old).

## Discussion

Concept elicitation by qualitative interviews promotes engagement and valuable inputs from the target population, allowing individuals to state the significance and impact of these events on their lives before researchers introduce a standardized or predetermined list of AEs^19^ ^,^ ^24^. Data from the cognitive interviews reported here were autobiographical descriptions and exemplifications regarding all 16 AEs on the list. All participants were very cooperative, allowing us to explore beyond the four AEs initially stipulated.

Adolescents tended to immediately identify physical symptoms, such as nausea, vomiting, and pain, as the most troublesome AEs related to chemotherapy regimens. Although this study did not analyze the prevalence of AEs, our findings corroborate the literature on the subject, which indicates these physical symptoms as some of the most frequent and bothersome AEs for this population^25^ ^-^ ^27^. Moreover, the interviews allowed the researchers to analyze and to report the vocabulary that adolescents use to describe these events.

Notably, participants indicated precipitating factors to some AEs or described known classifications to nausea, such as “anticipatory nausea”, relating it to psychological mechanisms. Anticipatory nausea and vomiting precede the administration of chemotherapy, corresponding to 20-30% of cases of nausea and vomiting induced by chemotherapy and tending to aggravate with factors related to treatment, anxiety, or negative experiences^28^ ^-^ ^29^.

Similarly to other publications, this study found that chemotherapy was considered an aggravating factor for some AEs, such as constipation^29^. Among antineoplastic drugs, vinca alkaloids are the leading cause of this AE^10^ ^,^ ^27^. Besides its relationship with chemotherapy, constipation can also worsen in pediatric cancer patients due to lower fluid and food intake, reduced mobility, and the use of opioid analgesics^28^.

Diarrhea was related to significant discomfort and, in some cases, to abdominal pain. It also implied embarrassment from recurrent use of the bathroom or diapers, something emotionally impacting for an adolescent^30^ ^-^ ^31^. The main drugs associated with diarrhea in this age group are 5-Fluorouracil, Irinotecan, Capecitabine, and Docetaxel - some of them used by the participants in this study^10^ ^,^ ^28^. 

Participants mentioned treatment-related pain. Pain in adolescents receiving antineoplastic treatment is frequent, persistent, and of high-intensity levels^32^.

Adolescents described pain sites which corroborated similar studies, which indicated the upper and lower limbs, joints, abdomen, head, back, and oral and perianal regions as the main sites of pain related to chemotherapy^2^ ^,^ ^4^ ^,^ ^11^ ^,^ ^33^.

Mucositis was related to pain and difficulties in eating. Participants detailed the manifestation and pain intensity of the inflammation and associated it with suffering, as observed in other studies^34^ ^-^ ^36^. This detailing indicated participants’ ability to recall significant aspects of their experience with this event and, mainly, to characterize the various dimensions and different attributes of the AE, which is expected in studies of this nature^30^ ^-^ ^31^.

Similarly to two other studies with the same age-range population, our study identified chemotherapy-induced peripheral neuropathy (CIPN) in patients, who used “numbness” and “tingling” to describe their sensations^21^ ^,^ ^27^. Since this AE is less frequent and less bothersome in adolescents, many health professionals tend to underestimate its importance^32^.

Adolescents also mentioned indisposition and lack of energy, known as weakness, a frequent symptom in studies with this population^19^ ^,^ ^27^ ^-^ ^28^. Weakness is considered a physical symptom despite being related to fatigue, a constitutional symptom. Adolescents may have difficulty in differentiating tiredness (fatigue) from weakness since these AEs, although different, frequently co-occur^21^ ^,^ ^27^ ^,^ ^37^.

Tiredness was the most used term to describe fatigue caused by chemotherapy treatment, corroborating other studies^19^ ^,^ ^21^ ^,^ ^27^. Fatigue is considered one of the most prevalent AEs experienced by adolescents with cancer and encompasses physical, psychological, and cognitive aspects^28^. It is a complex AE that affects the quality of life of pediatric oncology patients^12^. As the literature reports, fatigue is also associated with depressive symptoms and behavioral changes^27^ ^,^ ^37^. Participants reported that fatigue affects their routine activities, corroborating another qualitative study with adolescents undergoing chemotherapy^4^.

The first study to examine the fatigue and quality of life of Brazilian adolescents showed that fatigue occurs similarly in Brazilian pediatric cancer patients and in patients from other countries, likely because of the similarity of treatment protocols and characteristics of the disease despite cultural and socioeconomic differences^38^.

We observed that interviewees tended to talk about emotions when asked explicitly about feelings, not spontaneously. A study with American adolescents showed that participants responded to psychological wellness questions separate from physical wellness questions, different from adults^19^. The mood of some study participants fluctuated between sadness and anger. The prevalence of mood changes in adolescents undergoing chemotherapy varies from 30 to 70% in the literature^28^. Sadness was also associated with other AEs, such as depression and alopecia. Moreover, changes in appearance had an emotional impact on participants, as observed in the literature^4^ ^,^ ^27^ ^-^ ^28^. Research cites emotional instabilities as very uncomfortable AEs for adolescents, affecting their social relationships (i.e., parents and friends)^28^ ^,^ ^37^. Some drugs, such as corticosteroids, can significantly change mood and irritability^28^. Other emotional symptoms identified corroborate the literature, including anxiety and depression^19^ ^,^ ^27^.

Adolescents’ notion of the duration of events was another relevant aspect of our results which future studies should consider to assess AEs in this population. Participants related the onset of events to the action of chemotherapeutics, specifying the duration of the events and fully describing the temporal relationship between the use of cytotoxic drugs, the manifestation and duration of symptoms, and adverse effects. They even established more complex relationships, such as relating the duration of events to the average time interval for cell recovery, which is 21 days - in most of the treatment protocols used -, and understanding the transience of the experience with these events. Regarding the duration of the events, our findings corroborate descriptions in studies including adolescents^19^ ^,^ ^21^ ^,^ ^23^.

As for the reference period, most of the instruments aimed at adolescents use one week or a seven-day recall period^3^ ^,^ ^19^ ^,^ ^21^ ^,^ ^23^. However, researchers still have difficulty in establishing this duration due to the co-occurrence of different AEs and the individual variations and differences in therapeutic protocols^23^. Defining the recall period is essential in self-report instruments, whether focusing on adult patients, adolescents, or children^19^.

Only one participant mentioned cough, apparently due to a pulmonary infection. In the context of chemotherapy, cough can be secondary to pulmonary fibrosis, induced by some antineoplastic drugs such as Busulfan, Cyclophosphamide, Methotrexate (MTX), Mitomycin, and FOLFOX protocol drugs (Folinic Acid, 5-FU, and Oxaliplatin). Some of these drugs are more used in adolescents than in children^28^.

Adolescents did not differentiate between inappetence and incapacity to eat or difficulty in eating. In Brazil, many people use the expression “*I can’t eat*” to refer to lack of appetite, not to difficulties in eating. Thus, although we validated the information with participants, part of them could have misunderstood it or referred to the simultaneity of these events.

Adolescents used the same terms as researchers to express themselves about the adverse events of chemotherapy. Most adolescents clearly understood what we asked them about. Moreover, the most valuable contributions obtained during data collection indicate that participants felt comfortable answering the questions and could thus provide helpful information about the AEs experienced during chemotherapy. In some cases, they spontaneously used more complex terms to name the AEs and clearly showed interest and readiness to contribute to the study. 

Notably, cancer therapy is becoming more complex with the emergence of new drugs and associated AEs. This study has implications for nurses, who have a valuable role in managing the AEs of chemotherapy and a privileged position of educating patients and families. Since pediatric nurses have invaluable proximity to patients, listening to and using children’s voices will advance these nurses’ ability to relieve stressful events, promoting children’s quality of life during and after treatment.

This pioneering study in Brazilian adolescents is also a potentially valuable step for considering the voice of this population in chemotherapy symptoms and adverse events reports. Further work is needed since we mainly aimed to explore the wording, the understanding, and possible correlated factors recalled by adolescents when asked about these events. The perception of these events differs due to individual aspects and subjectivity. Thus, only individuals themselves can overvalue or minimize the impact of a particular event on them, along with aggravating factors, associated suffering, or other related AEs^39^. This study also represents an essential step toward validating an innovative tool that will finally report the adverse events related to chemotherapy in Brazilian adolescents according to the language and culture of the country.

We must recognize some limitations of this study, common to other research with adolescents. Despite how dense an investigation is, it will not fully capture all the dimensions of the experience with these adverse events - nor did our study intend to. Other limitations refer to the heterogeneity in the clinical profiles of the participants, including type of treatment protocols adopted, disease stage, and previous experience with the effects of the therapy used, besides the different time periods of treatment.

## Conclusion

The results of this study showed that adolescents with cancer can self-report clinically relevant AEs related to chemotherapy according to their subjective experience. The qualitative approach of this research included the valuable input from the target population, as suggested by elicitation studies. Studies investigating pediatric cancer patients’ subjective experience with chemotherapy are essential to support self-report instruments. This study shows significant results for understanding how adolescent patients identify, describe, and name AEs by cognitive interviewing.

Some adolescents suggested other terms to name the AEs, but most were satisfied with the primary terms used by the researchers. Unexpectedly, some participants opted to use more complex terms. The words and terms adopted show how understanding adolescents’ comprehension and language is essential for the science of symptoms and to study adverse events of cancer treatment. Moreover, results presented remarkable contributions related to aspects unexplored in Brazilian Portuguese and in the country’s cultural context.

Regardless of the scenario considered, this study showed that adolescents can describe adverse events associated with chemotherapy. This could contribute to the design of assessment instruments focused on this population to prevent, detect, and manage these events appropriately, with no interruptions in treatment planning and improving the quality of life of these patients.
